# Can Millet Consumption Help Manage Hyperlipidemia and Obesity?: A Systematic Review and Meta-Analysis

**DOI:** 10.3389/fnut.2021.700778

**Published:** 2021-08-17

**Authors:** Seetha Anitha, Rosemary Botha, Joanna Kane-Potaka, D. Ian Givens, Ananthan Rajendran, Takuji W. Tsusaka, Raj Kumar Bhandari

**Affiliations:** ^1^Smart Food Initiative, International Crops Research Institute for the Semi-Arid Tropics (ICRISAT), Patancheru, India; ^2^Development Strategy and Governance Division, International Food Policy Research Institute (IFPRI), Lilongwe, Malawi; ^3^Institute of Food, Nutrition and Health, University of Reading, Reading, United Kingdom; ^4^Food Chemistry Division, National Institute of Nutrition (NIN), Hyderabad, India; ^5^Organization for Advanced and Integrated Research, Kobe University, Kobe, Japan; ^6^National Technical Board of Nutrition, Government of India (GoI), New Delhi, India

**Keywords:** millets, hyperlipidemia, cholesterol, triacylglycerol, lipid profile

## Abstract

Many health benefits of millets (defined broadly to also include sorghum) have been advocated, including their roles in managing and preventing diabetes; however, the effects of millets on hyperlipidemia (high lipid levels) have been underrecognized. A systematic review and meta-analysis were conducted to collate available evidence of the impacts of millets consumption on lipid profile, namely total cholesterol (TC), triacylglycerol, high-density lipoprotein cholesterol (HDL-C), low-density lipoprotein cholesterol (LDL-C), and very-low–density lipoprotein cholesterol (VLDL-C). The results from 19 studies showed that the consumption of millets for periods as short as 21 days to 4 months reduced levels of TC, triacylglycerol, LDL-C, and VLDL-C (*p*<0.01) by 8.0, 9.5, 10 and 9.0%, respectively. Four studies demonstrated that millets consumption brought TC and triacylglycerol levels to the normal levels (<200 and <150 mg/dl, respectively). Furthermore, upon consumption of millet-based meals, there was a 6.0% increase in the HDL-C 4.0 and 5.0% reduction in systolic and diastolic blood pressure, and 7.0% reduction in body mass index (BMI). This evidence, leads us to conclude that consumption of millets reduces hyperlipidemia and hence hypertension, and raises the levels of HDL-C (good cholesterol), which can be beneficial for managing the associated risk of developing hypertension and atherosclerotic cardiovascular diseases in future.

**Systematic Review Registration:** The protocol of this systematic review has been registered in the online registration platform called “research registry” with the unique identification number “reviewregistry1123.”

## Introduction

Cardiovascular disease (CVD) is one of the leading causes of morbidity and mortality globally, accounting for 30% of deaths (1) worldwide, in which blood lipid profile plays a major role. Elevated levels of total cholesterol (TC), low-density lipoprotein cholesterol (LDL-C), very-low–density lipoprotein-cholesterol (VLDL-C), and triacylglycerol lead to progression of CVD and an increase of mortality. Among these, LDL-C is a major modifiable risk factor for atherosclerotic CVD (2), whereas, some types of the high-density lipoprotein cholesterol (HDL-C) are considered “good” cholesterol. Therefore, it is important to look at the ratio of TC to HDL-C in assessing the risk of CVD (3, 4). Along with lifestyle management, it is also vital to focus on a diet that can lower the risk of CVD by regulating metabolism and managing the lipid profile.

In developing countries, cereals typically occupy a major portion of the nutritionally unbalanced plate, which tends to be dominated by milled rice, maize, and refined wheat providing readily available carbohydrates. In addition, the so-called “junk food” and other unhealthy foods made from refined flour and fatty ingredients introduce large amounts of saturated fats into the body which, combined with sedentary lifestyles, can worsen the health of individuals. Lipid metabolism can be severely affected in individuals who are pre-diabetic, have type 2 diabetes mellitus, or other metabolic syndromes. Therefore, diet-based interventions for diabetes and other metabolic syndromes should contain ingredients that have a low glycemic index (GI) and the potential to correct metabolic abnormalities, such as deleterious lipid metabolism.

Millets are recognized as smart foods (5) as they fulfill the criteria of being “good for you,” “good for the planet,” and “good for the farmer,” Millets are traditional nutritious staple foods which can diversify staples and the overall diet when introduced, thereby playing a key role in controlling the levels of lipids in blood, managing metabolic disorders, such as diabetes and hyperlipidemia, and reducing the potential risk of developing CVD.

A recent systematic review and meta-analysis of low GI millets (including sorghum) and their effects on managing and reducing the risk of developing type 2 diabetes shows that millets have a beneficial effect on various outcomes, such as the fasting and post-prandial blood glucose levels, insulin index, and glycated hemoglobin (HbA1c) marker levels (6). This ability to manage and prevent diabetes may also help toward the management of hyperlipidemia. However, further, studies are needed to understand the effects of the consumption of millets on blood lipid management. This systematic review and meta-analysis are aimed at collating science-based evidence from randomized controlled trials and self-controlled clinical trials conducted through dietary intervention and cross-sectional studies on use of millets and their effects on lipid profile.

**Review question:** Does consumption of millet(s)-based foods help in managing blood lipid profile compared with regular, non-millet diets?

## Methods

### Study Period

The systematic review and meta-analysis were conducted from October 2017 to March 2021. The protocol of this systematic review has been registered in the online registration platform called “research registry” with the unique identification number “reviewregistry1123.”

### Information Sources

The study used the 27-item Preferred Reporting Items for Systematic Reviews and Meta-analyses (PRISMA) checklist (7) for every step of data collection, extraction, and analysis. Only studies that were published in the English language were considered. The scoping study was conducted using PubMed and MEDLINE to check for any existing studies on this topic and whether there were any overlaps with the research question as per the guidelines of (8). Search engines including Google Scholar, Scopus, Web of Science, PubMed, and CAB abstract were used to identify studies relevant to the research question. Search results obtained through a search strategy and keywords (Table 1) were further screened for relevance of the study, completeness of information, and quality of research, based on inclusion and exclusion criteria. If, after collecting the full papers, any required data were missing, the authors of the papers were contacted and complete information was requested for use in the meta-analysis.

**Table 1:** Search strategy and keywords used to identify relevant papers.

**Number**	**Criteria and keywords used for search**
1	Boolean logic such as “AND,” “OR,” “NOT” were used.
2	Efficacy of millets on lipid profile. Replaced the word “millets” with the names of millets, such as “barnyard millet,” “foxtail millet,” “finger millet,” “pearl millet,” “proso millet,” “brown top millet,” “little millet,” “kodo millet,” “teff,” “job's tears,” “fonio”
3	Impact of consuming millets on lipid profile
4.	Efficacy of millets on total triacylglycerol levels in humans. Replaced the word “total triacylglycerol” with “cholesterol,” “LDL-C,” “HDL-C,” “VLDL-C”

### Inclusion Criteria

**Figure 1:**
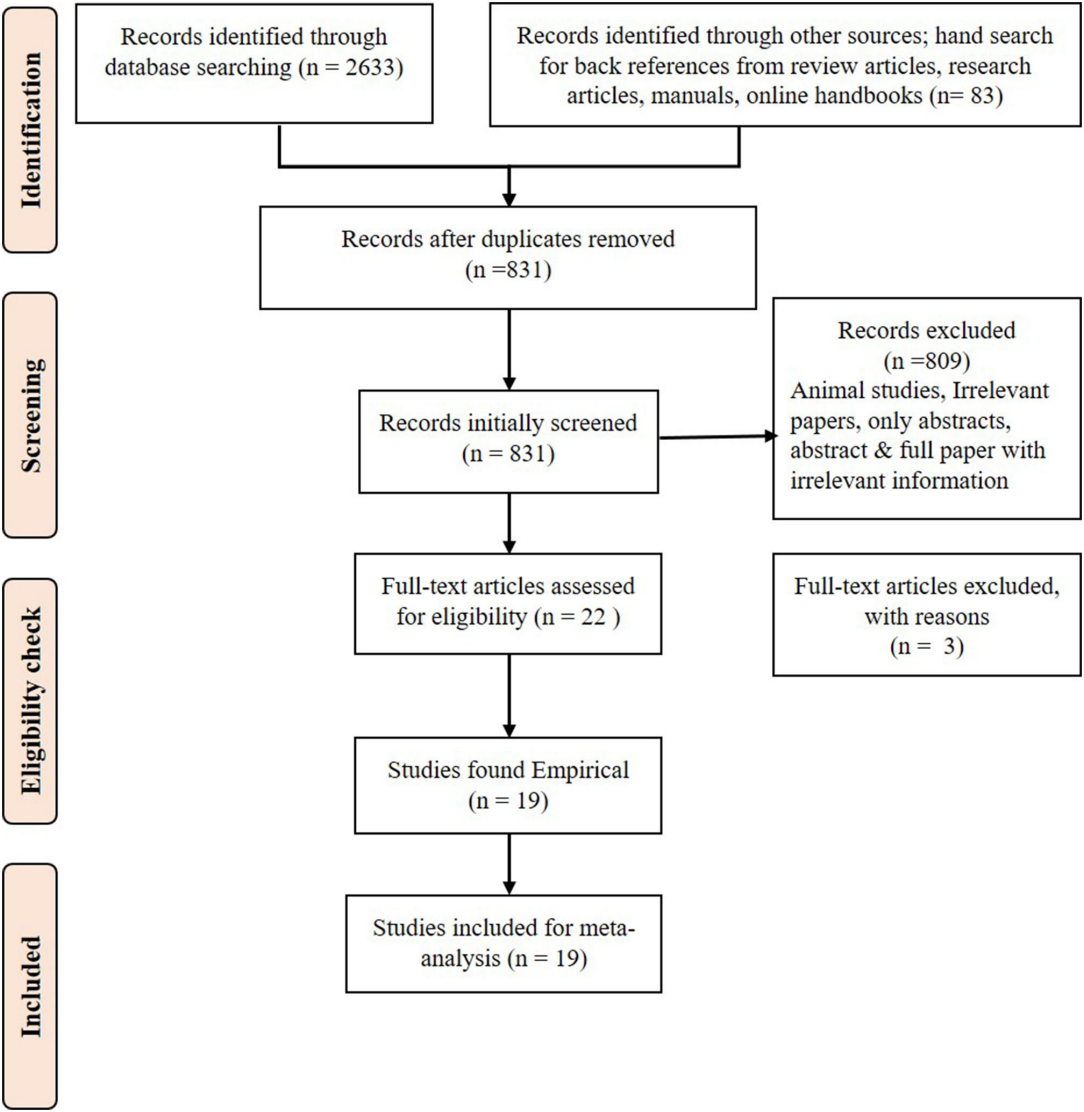
PRISMA flow diagram for systematic review.

The PRISMA flow diagram (Figure 1) shows the steps involved in the inclusion and exclusion of the studies. The articles published between 2011 and the first quarter of 2021, which fulfilled the criteria of being (1) Randomized controlled trials, self-controlled clinical trials, and/or cross-sectional studies conducted to test the efficacy of millets on TC, triacylglycerols, LDL-C, VLDL-C, and/or HDL-C; (2) studies that had information on any or all of the outcomes, including levels of TC, triacylglycerol, LDL-C, VLDL-C, and HDL-C; (3) studies on any age group or gender of any population to test the efficacy of millets on blood lipid profile; (4) studies that assessed the effects of millets on blood lipid profile along with or without weight, BMI, and systolic and/or diastolic blood pressure; and (5) only peer-reviewed journal articles were selected and included in the current systematic review.

### Exclusion Criteria

(1) Review articles were excluded from further consideration. (2) Animal studies were excluded. (3) In the case of incomplete data, the authors were contacted. If complete data were still not accessed, the study was excluded.

### Data Extraction

Each study was labeled with author details and year. The age group and gender of the participants were recorded along with the country, study method, sample size, type, and form of millets studied. The numerical variables considered for analysis included mean TC level, triacylglycerol, VLDL-C, LDL-C, and HDL-C in mg/dl, and weight gain or loss in kg. Systolic and diastolic blood pressure was recorded in mmHg. The data were then entered into Excel spread sheets as per guidelines provided by Harrer et al. (9). If standard error was recorded, it was converted to standard deviation (SD). Similarly, if the data were presented in different units, they were converted to the same unit to maintain uniformity. For example, cholesterol, and triacylglycerol concentrations given in mmol/L were converted to mg/dl to ensure the same unit of measurement is used for all the measurements.

### Quality Assessment of the Studies

Using the eight-item Newcastle–Ottawa Scale (10, 11), the quality of each study was assessed by two investigators, and any disagreement was resolved by discussing it with a third reviewer. The researchers also applied the principle of Bell et al. (12) to further strengthen the quality assessment.

### Risk of Bias Assessment

A funnel plot was used to assess publication bias if any. Other biases such as selection bias, detection bias, attrition bias, and reporting bias were assessed using the guidelines provided in Cochrane Handbook online version 6.2, (2021) for systematic reviews of interventions (13, 14).

### Summary Measures and Result Synthesis

Groups of individuals who were fed with millet-based meals for certain study periods (28 days to 4 months) were considered as intervention groups. The initial baseline measurement taken on these individuals was considered as the control measurement or pre-intervention measures, which consisted of regular rice- and wheat-based diets. Therefore, the before and after effects on primary outcomes, such as HDL-C, VLDL-C, LDL-C, TC, and triacylglycerol levels, and secondary outcomes such as weight, BMI, and systolic and diastolic blood pressure were included in the meta-analysis to measure the standard mean difference (SMD) and heterogeneity (*I*^2^). The significance of the results was determined using the fixed effect model for small samples, and/or single-source information with low heterogeneity among studies, and the random effect model for other cases (15, 16) and a *p*-value <0.05 indicates the significance of the effect. Results of both the fixed effect and random effect models were captured in each forest plot. A meta-analysis was conducted using the software R Studio version 4.0.4 (2021) to obtain forest plot along with heterogeneity (*I*^2^) and the overall test effect in the fixed effect and random effect models and funnel plots to determine publication bias (9, 17).

Descriptive statistics results such as mean, SD, and a percentage increase or decrease in TC, LDL-C, VLDL-C, HDL-C, triacylglycerol, weight, BMI, and systolic and diastolic blood pressure were calculated for both the intervention samples and control samples.

### Subgroup Analysis

Subgroup analysis was conducted for different types of millets used in the study.

## Results

Finger millet, foxtail millet, barnyard millet, and/or a mixture of millets (finger millet and little millet) were used in the 19 studies (18–36) that were included in one or more outcomes of meta-analysis. It was observed that consumption of millet-based food for a duration varying from 21 days to 4 months had a significant reducing effect on TC, triacylglycerol, VLDL-C, and/or LDL-C levels. Two cross-sectional studies included in the systematic review documented millet consumption information for up to 2 years. The consumption of millets was either in the form of biscuits, burfi (sweet), porridge, buns, boiled with water (similar to rice), roti (flatbread), dumpling, or upma. The amount of millets provided to the intervention groups varied from 50 to 200 g (dry weight basis) either in one meal or divided into two meals per day.

**Figure 2:**
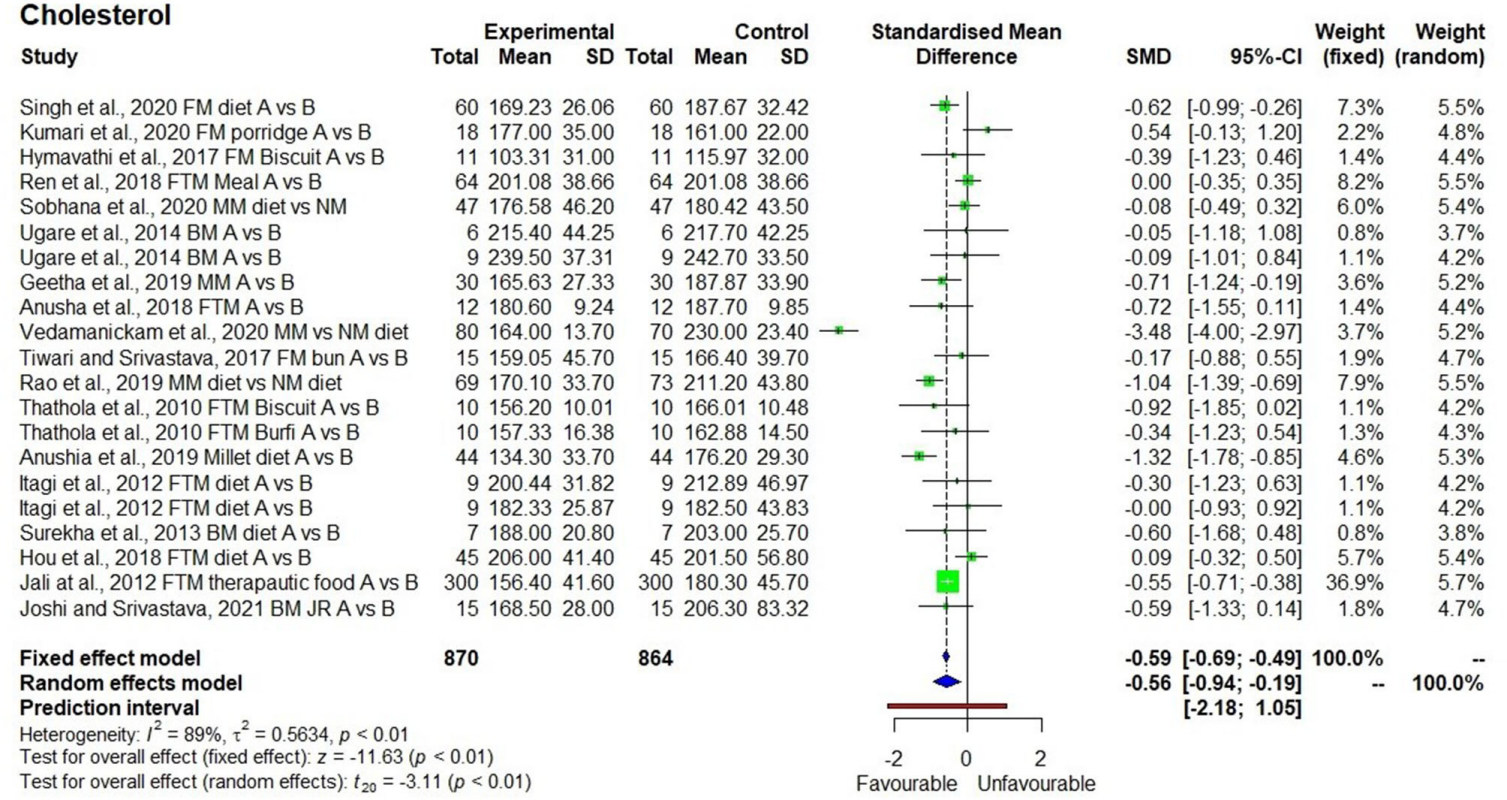
Effect of consuming millets on total cholesterol level (pre- vs. post-treatment or intervention vs. control diet).

The meta-analysis conducted on an outcomes-generated forest plot shows a significant reducing effect in TC levels after the consumption of a millet-based diet for 21 days to 4 months (*p* = 0.01) with SMD of −0.56, 95% class interval of −0.94 to −0.19, and *I*^2^ value of 89% confirming the heterogeneity (Figure 2). Descriptive statistics conducted for 870 samples, and 21 observations from the 18 studies showed that the TC levels decreased by 8% from 189.5 ± 27.7 to 174.8 ± 28.9 mg/dl. Three studies demonstrated the reduction in TC levels from 215.8 ± 12.5 mg/dl (>200 mg/dl hence hypercholesterolemia) to 167.5 ± 3.2 mg/dl (<200 mg/dl hence normal).

**Figure 3:**
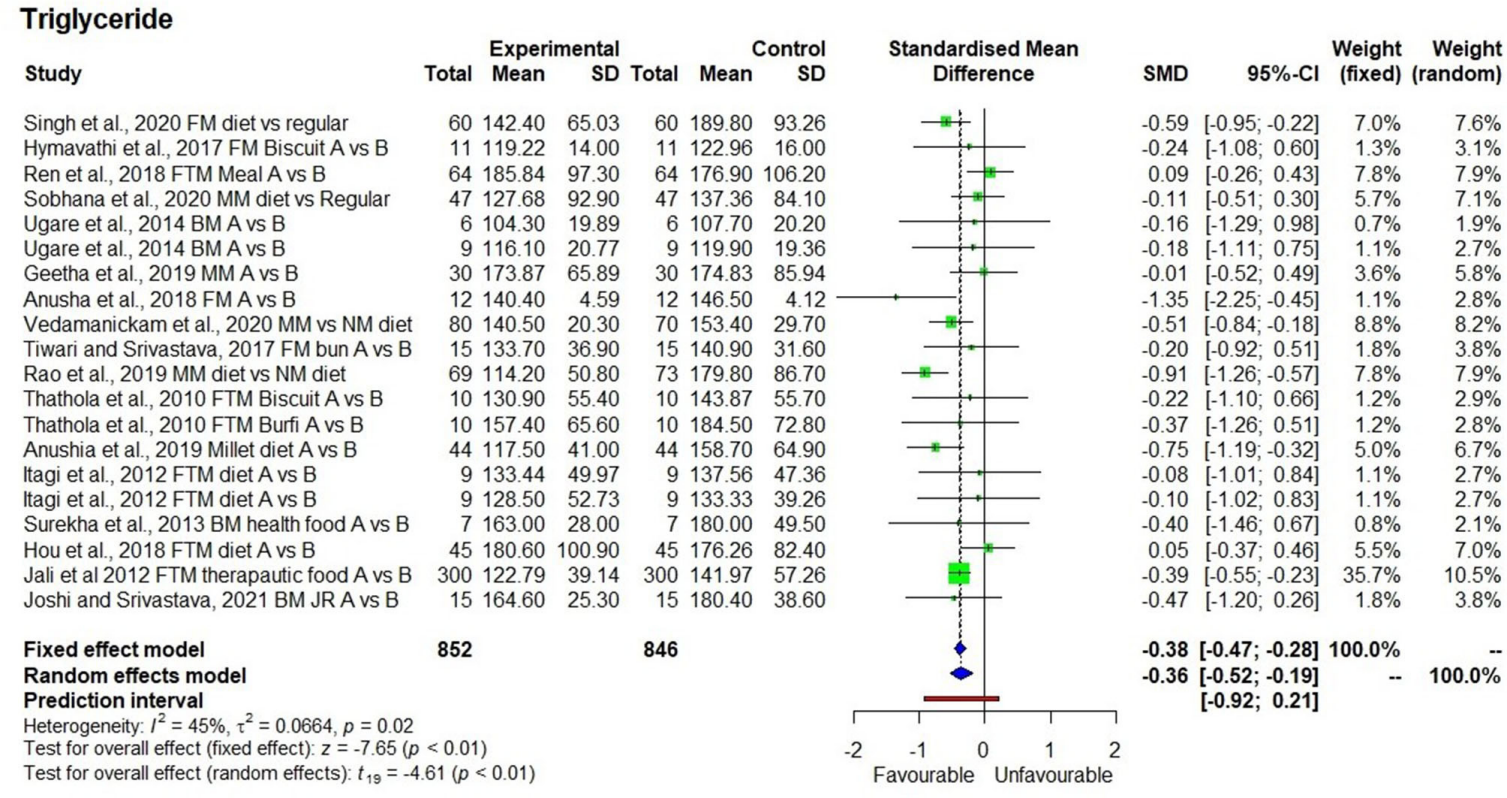
Effect of consuming millets on triacylglycerol level (pre- vs. post-treatment or intervention vs. control diet).

The consumption of millets for a period of 21 days to 2 years had a significant reducing effect on (*p* < 0.01) triacylglycerol levels with SMD of −0.38 and 95% confidence interval (CI) of −0.47 to −0.28. As the heterogeneity was low (*I*^2^ = 45%), the fixed effect model was used to interpret the results (Figure 3). Nonetheless, in both the fixed effect and random effect models, the effects were significant (*p* < 0.01). Descriptive statistics for 852 samples and 20 observations from the 17 studies showed a decrease in triacylglycerol levels by 9.5% from 154.3 ± 24.5 to 139.8 ± 23.5 mg/dl. There were four studies that demonstrated the reduction in hypertriacylglycerolemia (>150 mg/dl) to normal triacylglycerol levels (<150 mg/dl) after consumption of millets for 3 months with a mean reduction from 170.4 ± 17.2 to 128.6 ± 14.9 mg/dl (24.5% reduction) (18, 26, 28, 30).

**Figure 4:**
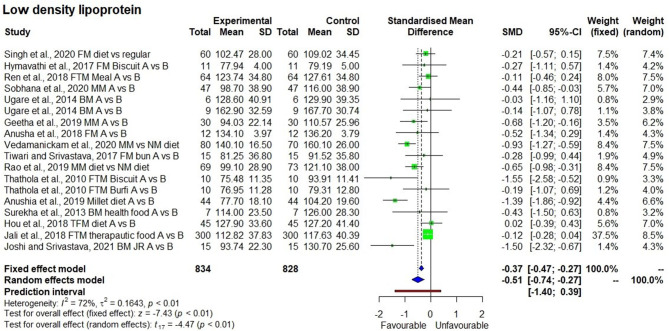
Effect of consuming millets on low-density lipoprotein-cholesterol (LDL-C) level (pre- vs. post-treatment or intervention vs. control diet).

The consumption of millet-based diets for long periods of time also had a significant reducing effect (*p* < 0.01) on LDL-C levels with *I*^2^ value of 72% (moderate heterogeneity among studies) with SMD of −0.51 and 95% CI of −0.74 to −0.27 (Figure 4). The LDL-C levels from 834 samples in 18 observations from the 16 studies were 118.2 ± 23.9 mg/dl, compared with 106.7 ± 25.4 mg/dl at the baseline (10% reduction). Particularly, five studies demonstrated the reduction in LDL-C from a moderately elevated level of 116.5 ± 10.0 to a normal level of 92.6 ± 8.7 mg/dl. One long-term cross-sectional study showed a reduction from the high level of LDL-C (160.1 ± 26 mg/dl) to the moderately high level (140.1 ± 16.5 mg/dl).

**Figure 5:**
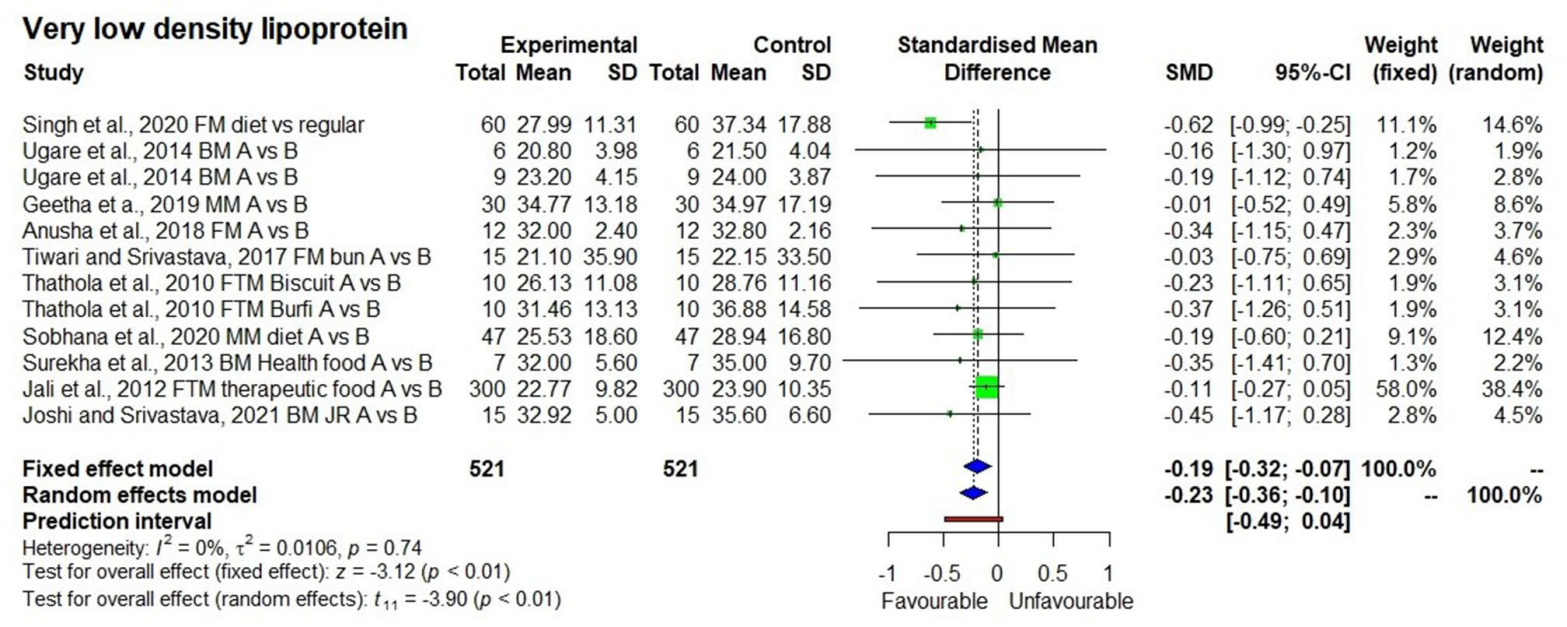
Effect of consuming millets on very-low–density lipoprotein-cholesterol (VLDL-C) level (pre- vs. post-treatment or intervention vs. control diet).

For VLDL-C meta-analysis, *I*^2^ value was 0% indicating no heterogeneity between the studies. However, both the fixed effect and random effect models showed a significant reducing effect in VLDL-C levels with *p*<0.01 (Figure 5). It is evident from 521 samples, 12 observations of the 10 studies that VLDL-C levels decreased by 9.0% from 30.2 ± 6.0 to 27.5 ± 4.9 mg/dl.

**Figure 6:**
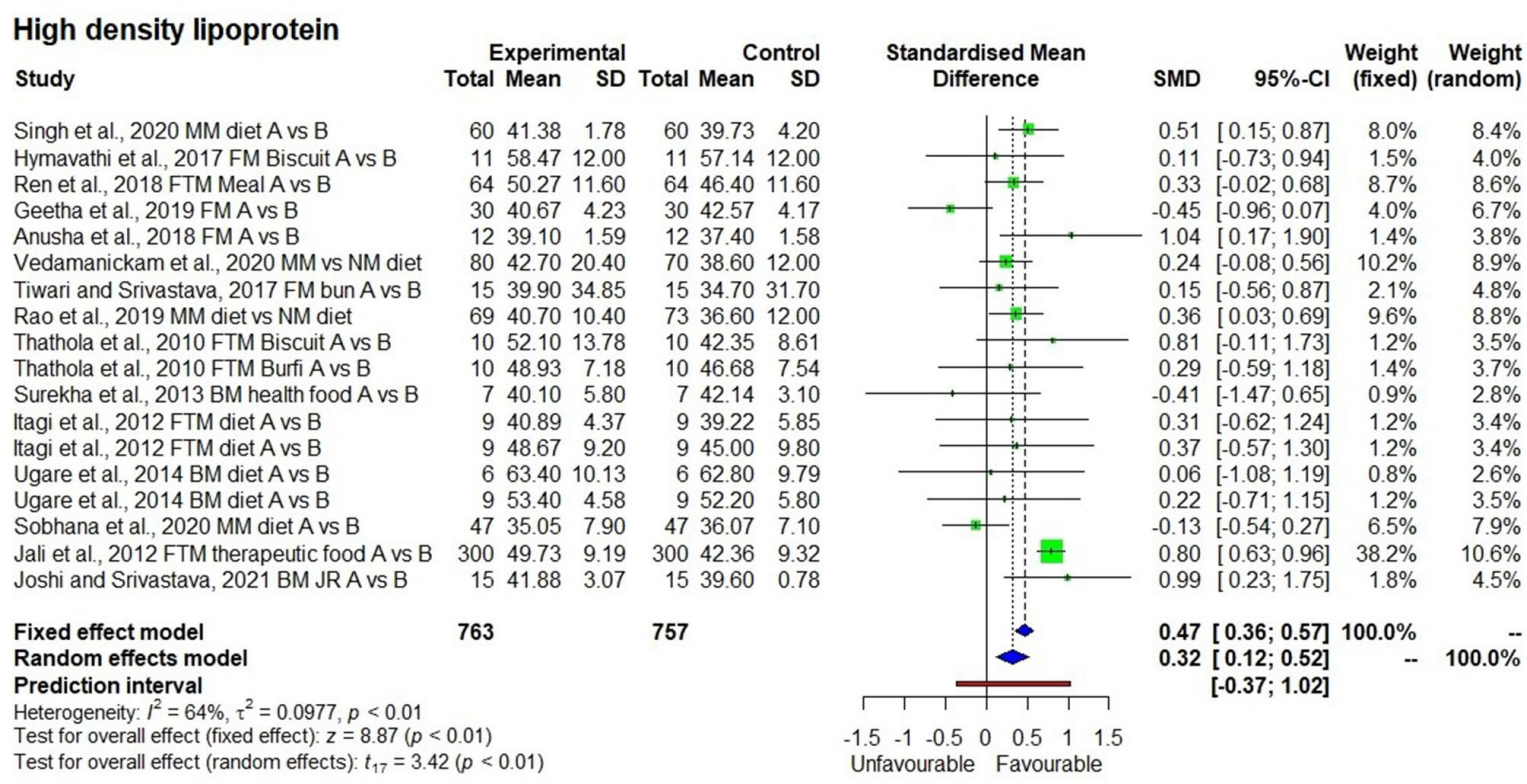
Effect of consuming millets on high-density lipoprotein-cholesterol (HDL-C) level (pre- vs. post-treatment or intervention vs. control diet).

In contrast to other outcomes, the HDL-C levels (Figure 6) had a significant increasing effect after consumption of millet-based foods for a long period (*p* < 0.01) with SMD of 0.32, 95% CI of 0.12–0.52. Descriptive statistics showed that HDL-C levels slightly increased (6.0%) from 43.4 ± 7.5 to 46.0 ± 7.6 as found in 763 samples in 18 observations from the 15 studies.

The ratio of TC to HDL-C was kept below 5 (ideal) by consuming barnyard millet–based meals for 28 days to two months (32, 35), where the TC:HDL-C ratio was reduced from 5.1 ± 0.14 to 4.2 ± 0.26.

**Figure 7:**
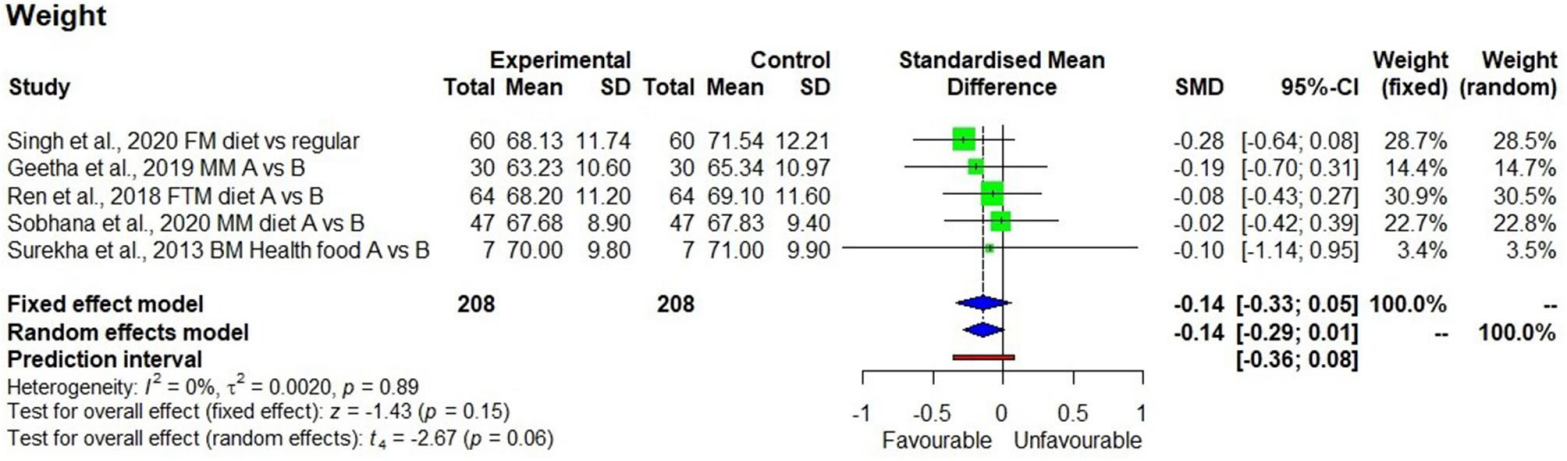
Effect of consuming millets on weight of the participants (pre- vs. post-treatment or intervention vs. control diet).

**Figure 8:**
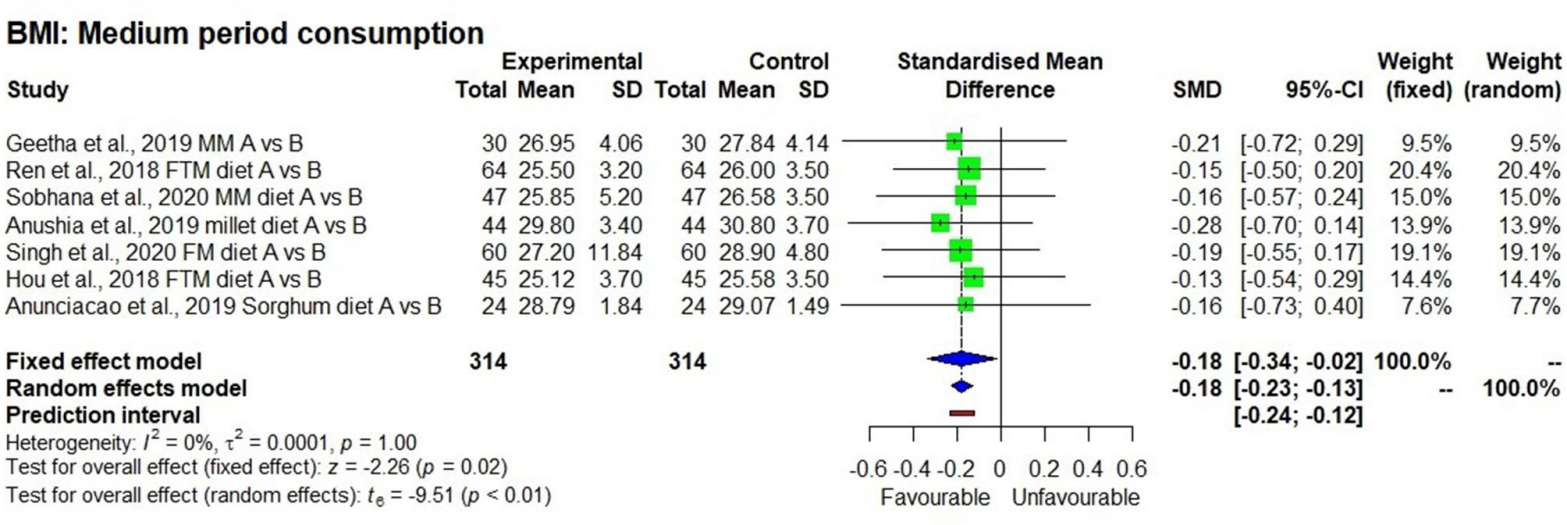
Effect of consuming millets on BMI of the participants (pre- vs. post-treatment).

**Figure 9:**
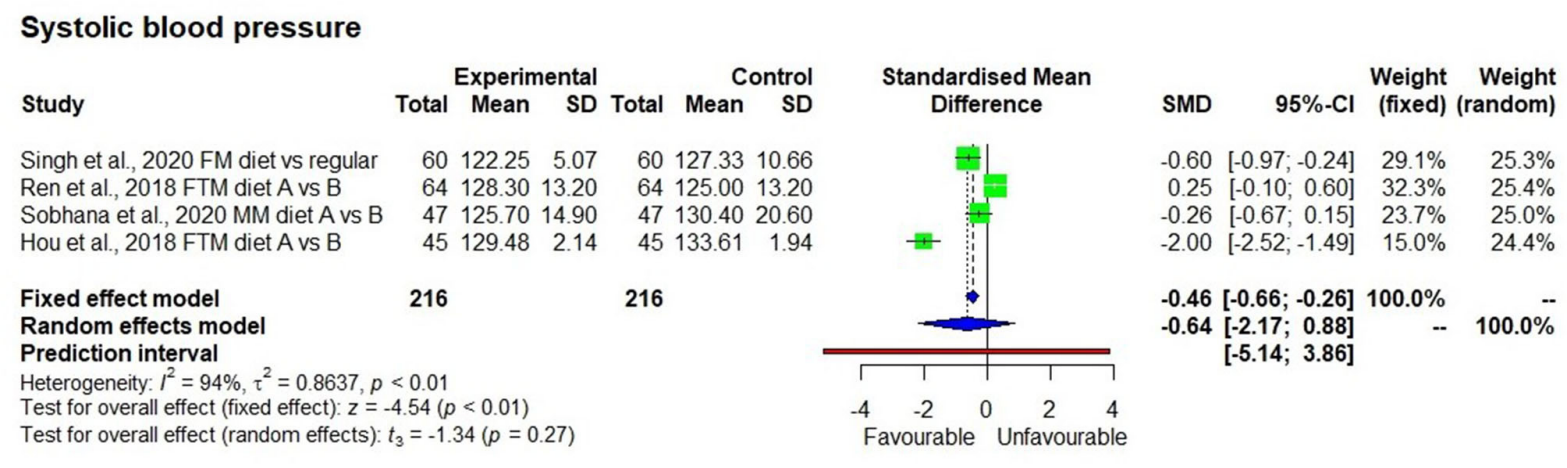
Effect of consuming millets on systolic blood pressure of the participants (pre- vs. post-treatment or intervention vs. control diet).

**Figure 10:**
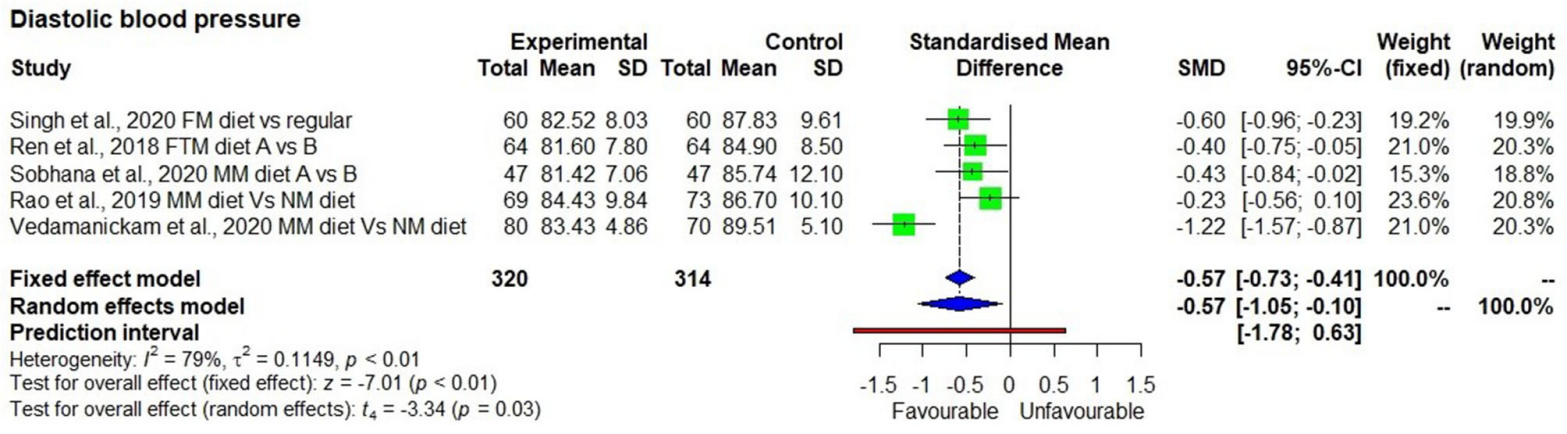
Effect of consuming millets on diastolic blood pressure of the participants (pre- vs. post-treatment or intervention vs. control diet).

Although, descriptive statistics showed a mean reduction in weight by 1.5 ± 0.4 kg among 208 study participants who consumed the millet-based diet, the effect on reduction was not statistically significant in a meta-analysis (*p* = 0.15). There were only four studies that measured systolic blood pressure, which showed a significantly reducing effect after consuming a millet-based diet (*p* < 0.01). Diastolic blood pressure showed a significantly reducing effect after consumption of millet-based diet (*p* < 0.01). BMI had significantly reducing effect by 7.0% after consumption of millet-based diets for 3–4 months (*p* < 0.05) with no heterogeneity observed among the studies (*I*^2^ = 0%) and 95% CI of −0.34 to −0.0.02 (Figures 7–10).

Publication bias was assessed using the funnel plot, and the observed asymmetry was adjusted using the trim and fit model to account for the small sample size effect until symmetry was achieved (*p* < 0.0001).

## Discussion

Hypertension, hyperlipidemia, diabetes, and smoking are the main risk factors for atherosclerotic CVD (37, 38). A recent systematic review on the effects of millets in managing and reducing the risk of developing type 2 diabetes showed that millets have beneficial effects on diabetes by providing low GI meals, reducing fasting and post prandial blood glucose levels as well as the glycated hemoglobin level (HbA1c) (6). The current systematic review focused on the effect on blood lipids as another important beneficial outcome of millet consumption. The meta-analysis showed that the ingestion of low (46.7 ± 12.0%) GI millet-based food had significant reductions in the levels of TC, triacylglycerol, LDL-C, and VLDL-C. Among other factors, the levels of glucose (and other simple carbohydrates) and saturated fats, and inappropriately controlled diabetes and metabolic syndromes are the causes of hyperlipidemia (37). Millets, being a low-GI food (6), reduce the blood glucose available for the synthesis of triacylglycerols (27). Moreover, millets also reduced VLDL-cholesterol which is a carrier of triacylglycerol in plasma, thereby it further reduced the triacylglycerol levels. This implies that millets play a key role in reducing triacylglycerol levels.

Overall, the 17 studies demonstrated an average of 9.5% reduction in triacylglycerol levels, and particularly four of the studies (18, 26, 28, 30) demonstrated that the plasma triacylglycerol levels reduced from a hypertriglyceridemic (>150 mg/dl) condition to normal (<150 mg/dl) when a millet-based meal was consumed once a day for 3 months instead of the regular rice- and/or wheat-based diets. Although, two cross-sectional studies that recorded more than 2 years of millet consumption showed that there was a reduction (26, 28), further, long-term randomized controlled trials would be necessary to confirm this.

LDL-C is considered as a major risk factor for CVD (39). Even with medication, residual LDL-C is associated with the risk of CVD. In the current study, consuming millets was shown to significantly reduce the LDL-C levels, and long-term consumption resulted in efficient reduction (26), indicating that long-term consumption of millets can help manage LDL-C levels and reduce the risk of CVD.

Reduction in the levels of LDL-C, VLDL-C, triacylglycerol, and TC is associated not only with the consumption of low-GI millets that produce a low glucose response and reduce the availability of glucose for triacylglycerol formation (27), but also with the consumption of unsaturated fatty acids from millets. The content of unsaturated fatty acids (both mono- and poly-unsaturated) is 2–10 times higher in millets compared with refined wheat and milled rice (Table 2). Some millets have 2.5, 5.7, and 7.8 times more unsaturated fatty acids, especially poly-unsaturated fatty acids compared with milled rice, whole wheat, and refined wheat. Intake of unsaturated fatty acids helps maintain high HDL-C levels. In addition, intake of poly-unsaturated fatty acids, which are essential in our diet, helps in lowering LDL-C levels and thereby lowering the risk associated with CVD (41, 42). Therefore, it suggests that replacing milled rice and refined wheat flour with millets bearing low GI and high poly-unsaturated fatty acids will help in reducing the risk of CVD. In this systematic review, there was 6.0% increase in HDL-C levels after consumption of millet-based meals as compared with regular rice or wheat meals. Thus, there is a need for further long-term randomized controlled trials to assess the impacts on HDL-C levels over an extended period of time. It is also noted that the refining process of teff, a type of millet, decreases the unsaturated fatty acid content and increases the risk of CVD (43).

**Table 2:** Fatty acid profile of the millets in comparison to other staple foods.

**Type of grain**	**Linoleic acid^a^**	**Oleic acid^b^**	**Mono-unsaturated fatty acids**	**Poly-unsaturated fatty acids**	**Saturated fatty acids**
	**(mg/100 g)**	**(mg/100 g)**	**(mg/100 g)**	**(mg/100 g)**	**(mg/100 g)**
Finger millet	362 ± 15	585 ± 36	585 ± 36	431 ± 21	317 ± 17
Pearl millet	1844 ± 57	585 ± 36	1047 ± 40	1984 ± 55	875 ± 35
Sorghum	508 ± 18	314 ± 14	314 ± 40	524 ± 18	163 ± 6
Kodo millet	576 ± 18	291 ± 7	297 ± 7	597 ± 18	246 ± 2
Little millet	1230 ± 43	868 ± 24	868 ± 24	1277 ± 48	589 ± 40
Maize, dry	1565 ± 18	700 ± 18	706 ± 17.4	1606 ± 18.5	413 ± 5.6
Rice, raw milled	234 ± 46	109 ± 21	117 ± 6.6	253 ± 13.2	184 ± 8.9
Wheat flour, refined	325 ± 7	51 ± 3	51 ± 3	343 ± 8	99 ± 2
Wheat flour, whole	697 ± 20	149 ± 8	149 ± 8	742 ± 19	206 ± 8

*Source: Longvah et al. (40)*.

a *Essential omega-6 fatty acid, beneficial for cardiovascular health, metabolism, and immune functions*.

b *A non-essential mono-unsaturated omega-9 fatty acid*.

Diastolic hypertension is common in individuals with components of the metabolic syndrome such as diabetes and hyperlipidemia, and diastolic blood pressure is the best predictor for future risk of CVD (44, 45). Five studies (18, 21, 22, 26, 28) showed that the average diastolic blood pressure in 320 subjects was slightly higher (86.9 ± 1.8 mmHg) than normal (<80 mmHg) at the beginning of the intervention, which was later decreased by 5% (from 86.9 ± 1.8 to 82.7 ± 1.3 mmHg), along with a decrease in lipids, after consuming a millet-based diet for 3 months compared with a regular rice/wheat-based diet. This suggests that consumption of a millet-based diet and diversification of staples with millet will help in reducing the risk of hypertension and associated CVD.

Obesity is a major concern as it raises the risk of CVD and type 2 diabetes. There was no statistically significant reduction in weight observed in the meta-analysis (*p* = 0.15), although, there was a reduction in the mean weight by 1.5 kg in 3 months. This suggests that more long-term studies with a larger sample size are needed for further investigation. Moreover, not all the studies that reported BMI also reported the actual weight.

On the other hand, it was evident from eight studies that on average there was a 7.0% reduction in BMI (28.5 ± 2.4 to 26.7 ± 1.8 kg/m^2^) in initially overweight and obese people, showing the possibility of returning to a normal BMI range (<25). Among the eight studies, six were randomized controlled trials conducted for 3–4 months, whereas, two others were cross-sectional studies conducted for 1–2 years. In the randomized controlled trials, there was no heterogeneity (*I*^2^ = 0.0%) observed between the studies, which could be due to the smaller number of studies, sample size, and the influence of geographical distribution (Asian population, mostly Indian). Moreover, there was no variation in effect size, with SMD of −0.18 for both fixed effect and random effect models. The result was significant both in fixed effect and random effect models (*p* < 0.05). Therefore, it was evident that millet-based food consumption can help reduce the degree of overweight and obesity (>30 BMI). In the open-label, self-controlled clinical trial by Ren et al. (21), they observed that a reduction in BMI to be associated with a significant reduction in body fat mass (from 22.1 ± 7.1 to 21.1 ± 7.2 kg) within 12 weeks of foxtail millet consumption compared with only regular diet with no significant reduction in body muscle mass, indicating that consumption of millet was particularly targeting the fat in the body. Another study also showed a significant reduction in body fat mass from 21.6 ± 0.96 to 20.92 ± 0.98 kg by consuming whole foxtail millet-based meals for 12 weeks compared with a regular diet (33).

Furthermore, studies also showed that consumption of millet-based foods caused satiety (32). Ren et al. (21) showed that consumption of foxtail millet for 12 weeks increased the blood leptin levels significantly (from 8.3 ± 6.4 to 9.6 ± 7.0 ng/ml), which is the indication of hunger suppression and reduced energy intake by altering the nervous system signals and blood glucose metabolism. The satiety is also due to slower gastric emptying time (32, 46, 47) and the high fiber content of millet (40). This characteristic may reduce sudden spikes in blood glucose levels, leading to decreased availability of glucose for triacylglycerol synthesis, thereby reducing triacylglycerol levels. This would need detailed research to further investigate and quantify the variables and impacts.

Hyperlipidemia is associated with inflammation, leading to lipotoxicity and progression of CVD. Some markers that might contribute to endothelial dysfunction include leptin, interleukin-6 (IL-6), and adiponectin. Potential tools for risk assessment include serum high-sensitivity C-reactive protein (hs-CRP), fasting insulin, tumor necrotic factor-α (TNF-α), IL-6, leptin, and adiponectin (48). A study conducted on foxtail millet consumption for 12 weeks (18) showed a decrease in inflammatory marker IL-6 from 6.2 ± 9.4 to 4.8 ± 5.5 pg/ml and TNF-α from 2.6 ± 5.5 to 1.4 ± 0.5 pg/ml. Another study on finger millet consumption for 12 weeks (18) showed a significant reduction in IL-6 (from 4.9 ± 0.7 to 1.60 ± 0.5 mmol/l) (*p* = 0.000) and TNF-α (from 7.8 ± 1.2 to 3.9 ± 0.6 mmol/l) (*p* = 0.016). However, Sobhana et al. (22) demonstrated that consumption of a millet-based diet for 3 months did not significantly reduce hs-CRP (from 0.45 ± 0.078 to 0.43 ± 0.064 μg/ml), which is not a significant reduction. More long-term studies are recommended to examine this effect. However, the reduction in IL-6 and TNF-α suggests that millet consumption can reduce inflammation caused by hyperlipidemia and diabetes.

Two studies examined the impacts of millets consumption on plasma antioxidant capacity. Hymavathi et al. (20) demonstrated significant increases in the levels of antioxidants such as superoxide dismutase from 79 ± 11 to 82 ± 12 U/ml, and reduced glutathione (GSH) level from 10.9 ± 2 to 11.9 ± 2 U/L, suggesting that consumption of finger millet could suppress stress levels in both diabetic and normal individuals. Consumption of millets for 8 weeks increased the ferric ion–reducing antioxidant power (19) from 679.5 ± 120.3 to 763.9 ± 105.3 (*p* < 0.05) and Trolox equivalent antioxidant capacity from 296.9 ± 122.8 to 431.0 ± 55.4 (*p* < 0.05). In other words, antioxidant capacity improved in healthy individuals upon consumption of millets. Similar studies should be conducted on pre-diabetic, diabetic, and hyperlipidemic individuals to understand the effects of tissue damage from metabolic syndromes.

In this systematic review, although, studies examined the impacts of millet consumption on blood lipid profile, none of the included studies focused on millet consumption and its impact on hyperlipidemia in other related disease conditions, such as non-alcoholic fatty liver disease (NAFLD), which is predicted to become a major cause of liver-related morbidity and mortality by 2030 (49). Consumption of whole grains increases the intake of unsaturated fatty acids and dietary fiber, which enhances the fullness or satiety value, which, in turn, could have a beneficial effect on reducing energy intake and hyperlipidemia, and reducing the risk of CVD and NAFLD (49). Further, research on long-term millet consumption and its association with inflammatory markers and reduction in hyperlipidemia, especially in NAFLD individuals, would help to construct dietary plans to reduce risks of development and progression of NAFLD and CVD.

### Recommendations

Overall, it is recommended that millet-based diets be designed and then promoted for management and prevention of atherosclerotic CVD as well as weight management. It would be beneficial to diversify major staples with millets across Africa and Asia, because millets have nutritional and health benefits, such as low GI and high levels of several necessary micro- and macronutrients (e.g., iron, zinc, calcium, and protein). Additionally, millets are a “smart food”: not only “good for you” but also “good for the planet” and “good for the farmer,” i.e., environmentally sustainable and climate-smart, with a lower carbon footprint. Therefore, they should also be part of solutions for reforming the food systems. This will help contribute to a range of UN Sustainable Development Goals, such as Zero Hunger, Good Health and Well-being, Responsible Consumption and Production, and Climate Action.

Priority research to address the limitations of this study or build more evidence includes the following. (1) Only five millets were assessed, namely finger millet, foxtail millet, barnyard millet, sorghum, and little millet combined with other millets in a meal. There are other millets and a range of varieties that are grown globally and expected to bear similar effects on managing hyperlipidemia. It is thus important to generate evidence with the unstudied crops/varieties. (2) Out of the 19 studies, two were conducted in China, one in Sri Lanka, and one in Brazil. The remaining 15 studies were from India, alluding to a geographical limitation. Millets are grown and consumed in all inhabited continents, especially across sub-Saharan Africa and South Asia. It will be useful to generate further evidence in different sub-populations. (3) The duration for randomized controlled trials varied from 21 days to 4 months. Research over a year or two will provide useful evidence. (4) No study was conducted on the effects of processing and cooking of millets on the lipid profile. It is critical to establish such a linkage. (5) Only three studies determined TC to HDL-C ratio, which is a key marker for CVD. Therefore, it is recommended to include all relevant parameters while implementing dietary interventions and assessing the impacts on hyperlipidemia and CVD. (6) The number of products in each category (baking, boiling, etc) was very small in the current systematic review hence the effect of processing on lipid profile was not evaluated which could be considered in future research. (7) As millets were identified to reduce hyperlipidemia, millets are expected to have a role in reducing or preventing NAFLD, which has not been studied to date.

## Conclusions

The systematic review executed in this study provides a strong evidence that millet consumption can improve blood lipid profile and thus exerts beneficial effects on management and prevention of hyperlipidemia, reduction in high blood pressure, weight, and BMI as well as an overall reduction in associated risk of CVD. Millets should therefore be integrated into nutrition and health strategies, utilized to diversify staples across Africa and Asia, and promoted as broader solutions in food system reforms. Further, analysis, customized to the situation, should be undertaken to ensure that millets are appropriately integrated into initiatives for maximized effectiveness.

## Data Availability Statement

The original contributions presented in the study are included in the article/Supplementary Material, further inquiries can be directed to the corresponding author/s.

## Author Contributions

SA and JK-P: conceptualization, methodology, data extraction, and writing. SA, RB, and TWT: methodology, data extraction, analysis, and interpretation. DIG, AR, and RKB: methodology, risk assessment, writing, and reviewing the manuscript until final stage. All authors contributed to the article and approved the submitted version.

## Conflict of Interest

The authors declare that the research was conducted in the absence of any commercial or financial relationships that could be construed as a potential conflict of interest.

## Publisher's Note

All claims expressed in this article are solely those of the authors and do not necessarily represent those of their affiliated organizations, or those of the publisher, the editors and the reviewers. Any product that may be evaluated in this article, or claim that may be made by its manufacturer, is not guaranteed or endorsed by the publisher.
